# Robotic versus open extended cholecystectomy for T1a–T3 gallbladder cancer: A matched comparison

**DOI:** 10.3389/fsurg.2022.1039828

**Published:** 2022-11-07

**Authors:** Jun Yang, Enliang Li, Cong Wang, Shuaiwu Luo, Zixuan Fu, Jiandong Peng, Wenjun Liao, Linquan Wu

**Affiliations:** Department of General Surgery, The Second Affiliated Hospital of Nanchang University, Nanchang, China

**Keywords:** gallbladder cancer (GBC), extended cholecystectomy, robotic surgery, propensity score matching, surgical outcomes

## Abstract

**Background:**

The feasibility and safety of robotic extended cholecystectomy (REC) are still uncertain. This study was performed to compare the short- and long-term outcomes of REC with those of open extended cholecystectomy (OEC) for T1a–T3 gallbladder cancer.

**Methods:**

From January 2015 to April 2022, 28 patients underwent REC in our center. To minimize any confounding factors, a 1:2 propensity score-matching analysis was conducted based on the patients’ demographics, liver function indicators, T stage, and symptoms. The data regarding demographics, perioperative outcomes, and long-term oncologic outcomes were reviewed.

**Results:**

The visual analogue scale score was significantly lower in the REC than OEC group immediately postoperatively (3.68 ± 2.09 vs. 4.73 ± 1.85, *P* = 0.008), on postoperative day 1 (2.96 ± 1.75 vs. 3.69 ± 1.41, *P* = 0.023), and on postoperative day 2 (2.36 ± 1.55 vs. 2.92 ± 1.21, *P* = 0.031). In addition, the REC group exhibited a shorter time to first ambulation (*P* = 0.043), a shorter time to drainage tube removal (*P* = 0.038), and a shorter postoperative stay (*P* = 0.037), but hospital costs were significantly higher in the REC group (*P* < 0.001). However, no statistically significant difference was found in the operation time (*P* = 0.134), intraoperative blood loss (*P* = 0.467), or incidence of postoperative morbidity (*P* = 0.227) or mortality (*P* = 0.289) between the REC and OEC groups. In regard to long-term outcomes, the 3-year disease-free survival rate was comparable between the OEC and REC groups (43.1% vs. 57.2%, *P* = 0.684), as was the 3-year overall survival rate (62.8% vs. 75.0%, *P* = 0.619).

**Conclusion:**

REC can be an effective and safe alternative to OEC for selected patients with T1a–T3 gallbladder cancer with respect to short- and long-term outcomes.

## Introduction

Gallbladder cancer (GBC) refers to malignant tumors that occur in the gallbladder, including the base, body, neck, and cystic duct. In China, GBC accounts for 0.4%–3.8% of all biliary tract diseases and ranks sixth among all gastrointestinal cancers (1, 2). Moreover, the overall mean survival rate for patients with GBC is 6 months, and the 5-year overall survival rate is 5% (3). Radical resection is the only curative treatment for GBC (4). Therefore, the choice of surgical technique is particularly important, and whether to perform extended resection depends on the patient's preoperative imaging data and intraoperative frozen section results.

Extended cholecystectomy can greatly improve the postoperative survival time and quality of life of patients with GBC, but traditional open extended cholecystectomy (OEC) is associated with many postoperative complications (e.g., bleeding, bile leakage, and poor wound healing) that can lead to slow recovery and a prolonged hospital stay (5–7). However, since the inception of robot-assisted liver resection in 2003, robotic extended cholecystectomy (REC) has gained widespread acceptance (8). The development of robotic surgical systems has promoted treatment of GBC in the past decade, and the number of patients with GBC receiving REC has rapidly increased (9–11).

This study involved patients with stage T1a–T3 GBC according to the 8th edition of the TNM Classification of Malignant Tumors of the American Joint Committee on Cancer (AJCC) who were treated by extended hepatectomy with gallbladder resection (12). We retrospectively analyzed the clinical data of 28 patients with GBC treated with REC. To reduce the confounding bias, a 1:2 propensity score-matching (PSM) analysis was conducted in the REC and OEC groups. The perioperative data and follow-up results were analyzed to provide clinical evidence for more rapid recovery after robotic surgery than after open surgery for the treatment of GBC.

## Patients and methods

### Study design

**Figure 1:**
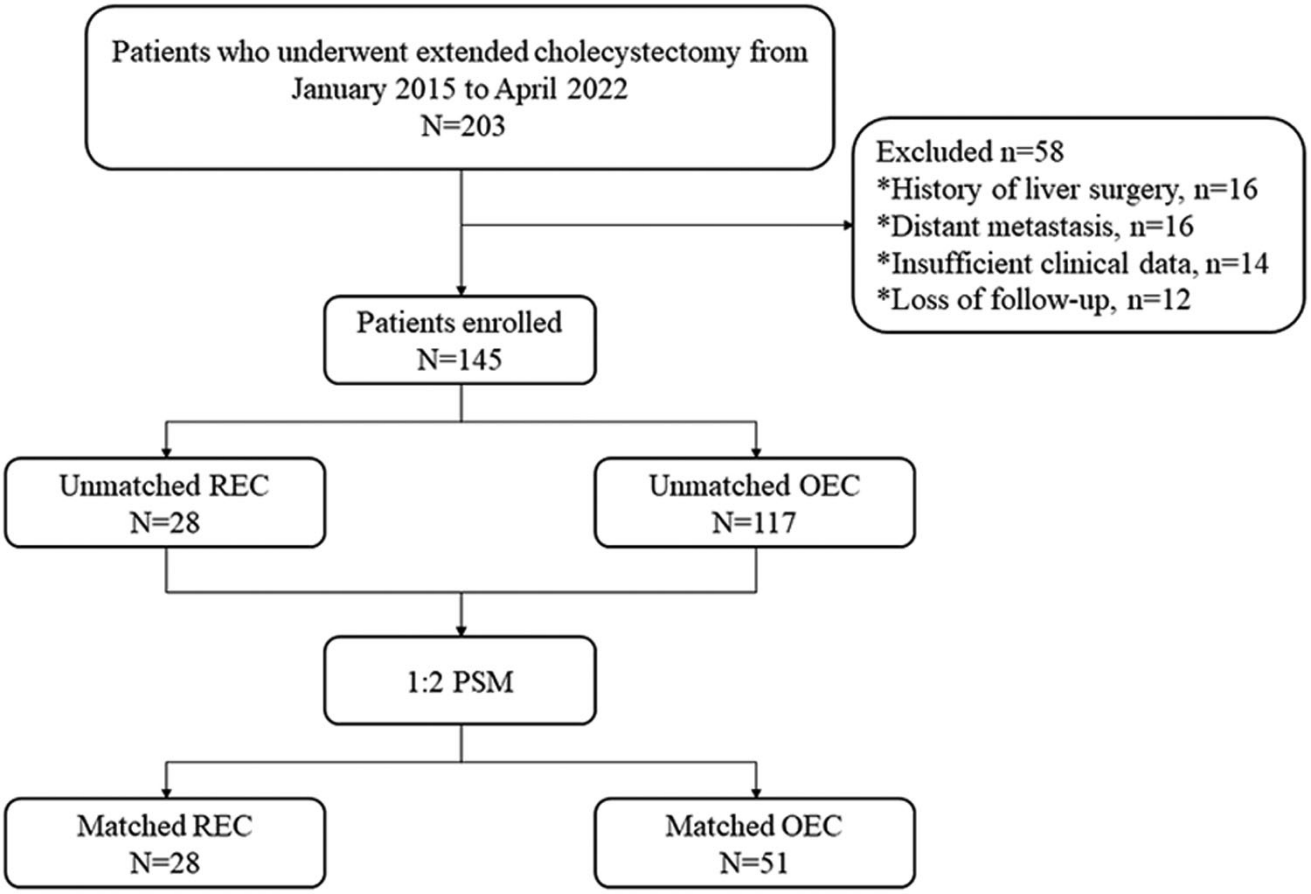
Flow chart of study design. REC, robotic extended cholecystectomy; OEC, open extended cholecystectomy; PSM, propensity score matching.

From January 2015 to April 2022, 28 consecutive patients who underwent REC for the treatment of GBC at the Second Affiliated Hospital of Nanchang University and met the inclusion criteria were analyzed. During the same period, 117 patients who underwent OEC for GBC were also included (Figure 1). All patients had undergone preoperative computed tomography (CT) or magnetic resonance cholangiopancreatography as well as a multidisciplinary consultation including surgery, medical oncology, hepatology, and imaging experts. Clinicopathological data were complete. Non advanced malignancy was identified by pre-operative multi-slice spiral enhanced CT and enhanced magnetic resonance imaging combined with cholangiopancreatography. Enhanced CT examination could show the extent of gallbladder wall invasion, whether adjacent organs were involved, and lymph node metastasis (13). Magnetic resonance cholangiopancreatography could clearly show the anatomical relationship of the pancreatic duct and determine whether there was biliary obstruction. Contrast-enhanced magnetic resonance imaging could identify tumor size, liver invasion, vascular invasion, abdominal lymph node metastasis and distant metastasis (14). Before the operation, all patients were also expected to achieve complete resection without combined resection of adjacent organs other than the liver (patients with distant metastasis were excluded). All patients were well enough to tolerate the operation under general anesthesia and had no history of abdominal surgery. The cases of robotic extended cholecystectomy converted to open surgery, suffered major vascular injuries and needed vascular reconstruction were eliminated. Therefore, 28 patients included in this study were not converted to open surgery, suffered major vascular injuries and needed vascular reconstruction. Because of the high cost associated with REC, REC was performed only in patients who voluntarily agreed to undergo robotic surgery after being fully informed of the differences between the conventional open and robotic approaches. The hospital costs of our medical center were composed of the following 9 aspects: cost for comprehensive medical services, diagnostic cost, treatment cost, rehabilitation cost, cost for traditional Chinese medicine, drug cost, cost for blood and blood products, cost for consumables and other cost. We used propensity scores to match patients in a 1:2 ratio according to age, sex, body mass index, albumin concentration, total bilirubin concentration, American Society of Anesthesiologists classification, alanine aminotransferase concentration, aspartate aminotransferase concentration, T stage, and symptoms. The study was conducted in accordance with the Helsinki Declaration of 1,964 and all subsequent amendments, and it was approved by the Ethics Committee of the Second Affiliated Hospital of Nanchang University in China. All patients provided written informed consent.

### Surgical procedures

To effectively demonstrate the surgical procedures of REC, the following text describes a representative case involving a 68-year-old man with stage T1b GBC according to the AJCC 8th edition staging criteria who underwent wedge resection around the gallbladder fossa.

#### Preoperative preparation and trocar locations

**Figure 2:**
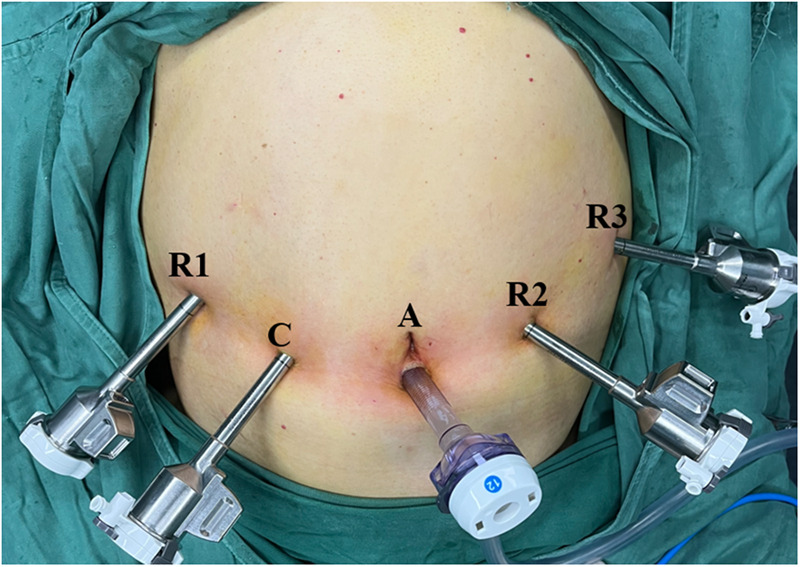
Photograph showing positions of the trocars: A, assistant port (12 mm); C, camera port (8 mm); R1, operation port 1 (8 mm); R2, operation port 2 (8 mm); R3, operation port 3 (8 mm).

The patient was placed in the lithotomy position under general anesthesia. The assistant surgeon stood between the patient's legs with the robot cart located over the patient's head. First, a 12-mm trocar was inserted immediately inferior to the umbilicus using the open technique. Carbon dioxide gas was infused into the intraperitoneal cavity until the pressure reached 14 mmHg. The intra-abdominal space was explored *via* video scope before the other trocars were inserted. The positions of the trocars are shown in Figure 2. These positionings were not absolute but varied instead according to the patient's body size and anatomy. We ensured the space of at least one fist between trocars to minimize interference among instruments. The patient was then placed in the reverse Trendelenburg position and slightly left side down. Robotic arms were docked to each trocar.

#### Dissection of Calot’s triangle and lymph nodes

**Figure 3:**
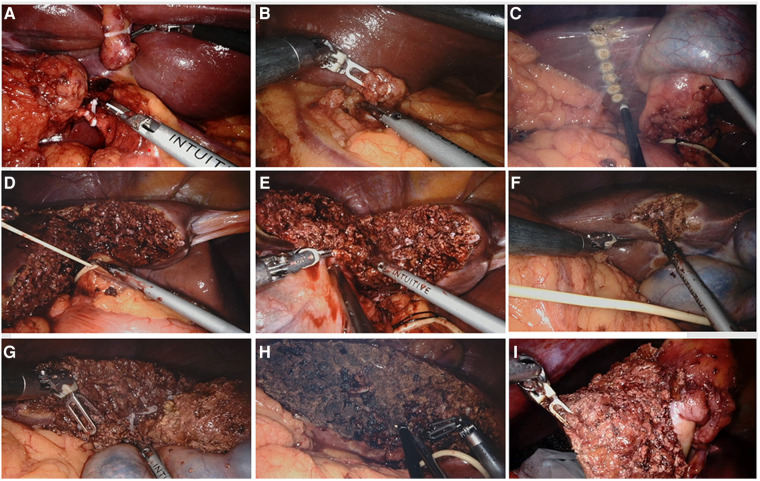
Surgical procedures of robotic extended cholecystectomy (**A**) ligation of cystic artery and cystic duct. (**B**) Regional lymph node dissection. (**C**) Hepatectomy line 2–3 cm around the gallbladder bed. (**D**) Blockage of hepatic portal. (**E**) Incision of liver tissue on left side of gallbladder along hepatectomy line. (**F**) Incision of liver tissue on right side of gallbladder along hepatectomy line. (**G**) Complete resection of liver tumor. (**H**) Electrocoagulation of liver wound for hemostasis. (**I**) Removal of specimen.

The left and right perihepatic ligaments and all loose connective tissues in the exposed area of the liver were removed so that the liver was completely free. Dissection then commenced from the right side of porta to the hilum to expose the right lateral wall of the bile duct and portal vein. Calot's triangle was dissected and the cystic duct ligated flush to the common bile duct (Figure 3A). Next, the supraduodenal region of the porta hepatis and hepatoduodenal ligament was dissected to expose the common hepatic artery. The superior border of the pancreas was exposed, and the common hepatic artery was identified. The adipose and connective tissues of the porta hepatis were sharply separated, the lymph nodes of station 12 (12a, 12b, and 12p) were dissected respectively, the adipose and connective tissues of the celiac trunk were sharply separated, and the lymph nodes of stations 8 and 9 were dissected (Figure 3B).

#### Liver resection

The transection plane was demarcated with electrocauterization on the liver surface, 2–3 cm from the gallbladder bed (Figure 3C). Controlled low central venous pressure technology and pre-indwelling hepatic portal block tape were routinely used during the operation to reduce the risk of hepatic vein bleeding (Figure 3D). The parenchymal dissection was carried out with a harmonic scalpel, beginning from the left side (Figure 3E). Visualized vessels and bile ducts were ligated by the clip applier, electrocauterization, or suturing. After the left-side liver dissection was nearly completed, dissection was performed on the right side of the transection line (Figure 3F). Finally, the inferior portion of the liver was dissected upward to complete the liver resection (Figure 3G). The specimen was placed in a specimen bag and set aside in the intra-abdominal space for removal at the end of surgery (Figures 3H,I). Liver wounds with active bleeding or bile leakage were treated with electrocoagulation rods or 4–0 Prolene sutures. After confirming the absence of active bleeding and bile leakage in the liver wound, a drain was placed around the resection plane and tagged on the retroperitoneum. The umbilical incision was extended by an additional 2–3 cm, and a wound protector was applied to prevent port site recurrence.

#### Surgical procedures of open extended cholecystectomy

For traditional surgical procedures of open extended cholecystectomy, an inverse L-shaped right subcostal incision was performed. First, Calot's triangle was dissected and the cystic duct ligated flush to the common bile duct. Next, the supraduodenal region of the porta hepatis and hepatoduodenal ligament was dissected to expose the common hepatic artery. The superior border of the pancreas was exposed, and the common hepatic artery was identified. the lymph nodes of stations 8, 9 and 12 (12a, 12b, and 12p) were dissected respectively. Finally, resecting liver tissue 2–3 cm around the gallbladder bed until completing resection of liver tumor.

### Perioperative care and follow-up

All patients in both groups underwent routine preoperative care, such as standard perioperative education, no eating or drinking for 8 h before surgery, and no preoperative bowel preparation or premedication. Postoperative complications were documented and graded according to the Clavien–Dindo classification. The follow-up protocol included a clinical examination, abdominal contrast-enhanced CT, and measurement of serum tumor markers (including carcinoembryonic antigen and carbohydrate antigen) every 3 months.

### Statistical analysis

Continuous data are expressed as mean ± standard deviation, and categorical variables are expressed as *n* (%). The Mann–Whitney *U* test was used to compare continuous variables, and Pearson's chi-square test was used to compare discrete variables. All analyses were performed using SPSS 26.0 software (IBM Corp., Armonk, NY, USA), and the “R-3.5.3-win” R package was used to perform the PSM analysis. The matching was performed in a 1:2 ratio, and a caliper width of 0.2 standard deviations was specified. Kaplan–Meier estimates for overall survival (OS) and disease-free survival (DFS) were compared between the OEC group and the REC group using the log-rank test. A *P* value of <0.05 was considered statistically significant.

## Results

### Clinical characteristics of 145 eligible patients before PSM

**Figure 4:**
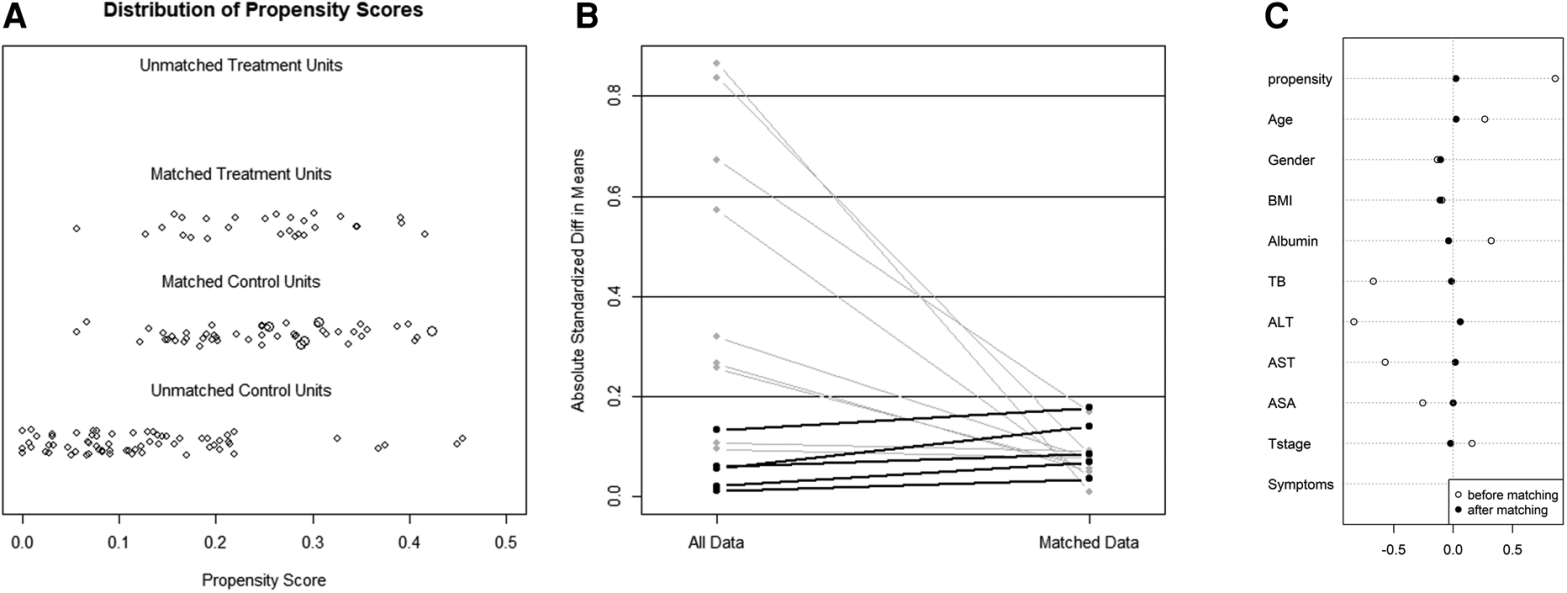
The model values of the SMD before and after propensity score-matching analysis. (**A**) The unmatched robotic extended cholecystectomy (treated) and open extended cholecystectomy (control) data were removed, and the matched treatment and control data were preserved. (**B**) The SMD of the propensity score and 10 confounders before and after propensity score matching is depicted in a line plot. (**C**) The SMDs of the propensity score and 10 confounders (age, sex, BMI, albumin, TB, ALT, AST, ASA, T stage, and symptoms) are depicted as hollow dots, and the SMDs of the matched data are depicted as solid dots. SMD, standardized mean difference; BMI, body mass index; TB, total bilirubin; ALT, alanine aminotransferase; AST, aspartate aminotransferase; ASA, American Society of Anesthesiologists classification.

**Figure 5:**
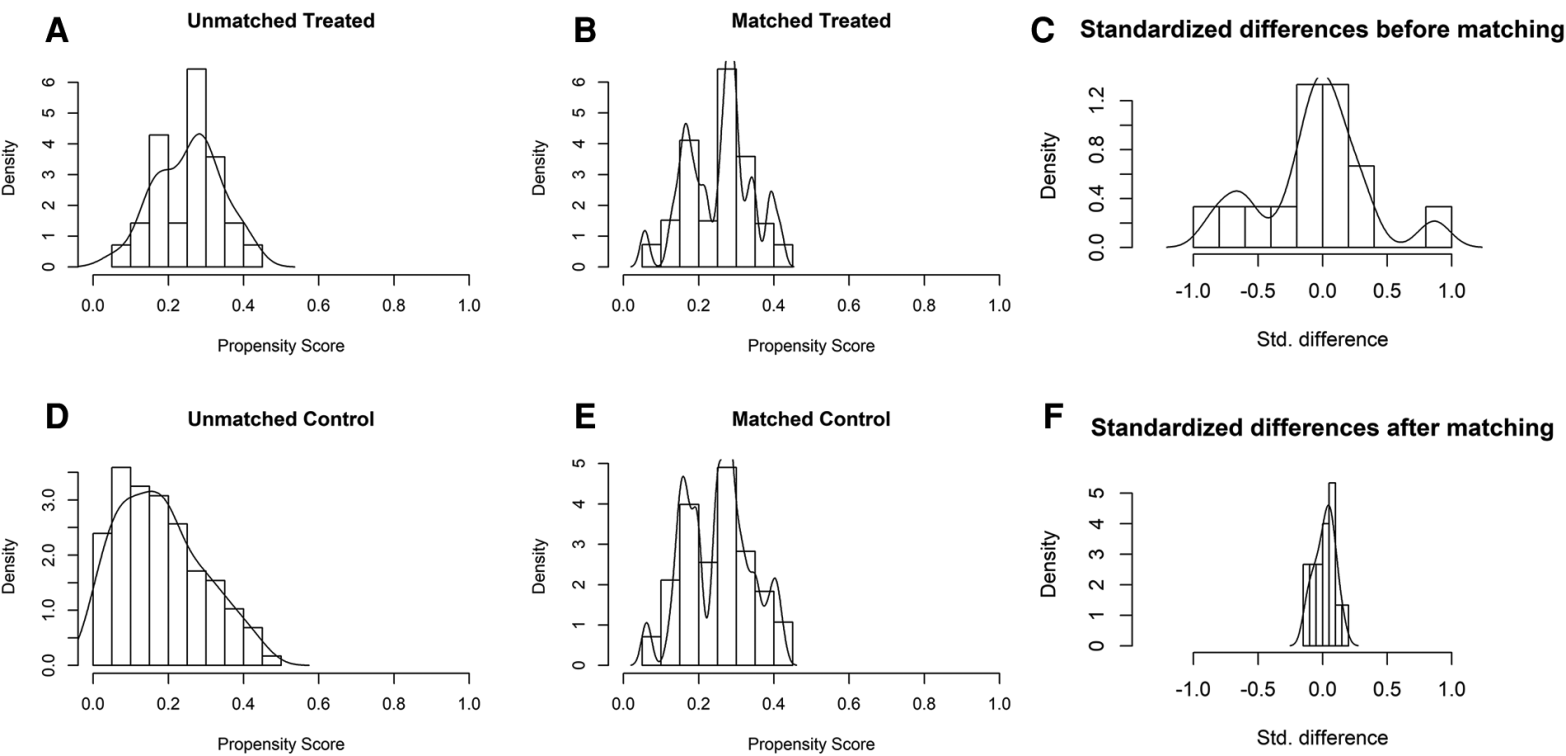
Distribution of propensity scores of robotic extended cholecystectomy (treated) and open extended cholecystectomy (control) (**A,D**) before and (**B,E**) after matching with overlaid kernel density estimate. Histograms with overlaid kernel density estimates of standardized differences (**C**) before and (**F**) after matching.

Of the 145 eligible patients diagnosed with GBC, 28 (19.3%) underwent REC and 117 (80.7%) underwent OEC. The clinical characteristics of the two groups are shown in Table 1. There were significant differences in the total bilirubin, alanine aminotransferase, and aspartate aminotransferase concentrations (*P* = 0.044, *P* = 0.019, and *P* = 0.017, respectively) before PSM as a result of a conspicuous bias with the pre-described propensity scores (REC group vs. OEC group: 0.254 vs. 0.179, *P* < 0.001) (Figures 4, 5).

**Table 1:** Patient characteristics according to operation type by unmatched and matched data.

Variables	Unmatched data			Matched data		
	Control (*n* = 117)	Treated (*n* = 28)	*P*-value	Control (*n* = 51)	Treated (*n* = 28)	*P*-value
Age (years)	55.27 ± 14.10	58.50 ± 12.15	0.257	57.08 ± 13.82	58.50 ± 12.15	0.610^a^
Gender (male/female)	59/58	16/12	0.523	29/22	16/12	0.981^a^
BMI (kg/m^2^)	25.08 ± 3.81	24.75 ± 3.48	0.279	24.21 ± 3.77	24.75 ± 3.48	0.364^a^
Albumin (g/L)	36.61 ± 4.37	38.04 ± 4.48	0.968	38.06 ± 3.40	38.04 ± 4.48	0.205^a^
TB (μmol/L)	29.79 ± 30.75	23.22 ± 9.76	0.044*	22.21 ± 14.7	23.22 ± 9.76	0.261^a^
ALT (U/L)	65.54 ± 66.84	42.79 ± 27.18	**0**.019*	43.39 ± 27.39	42.79 ± 27.18	0.415^a^
AST (U/L)	74.57 ± 92.96	52.31 ± 38.84	**0**.017*	45.17 ± 30.12	52.31 ± 38.84	0.854^a^
ASA						
≤2	95	25	0.309	47	25	0.668^a^
>2	22	3		4	3	
T stage						
T1a	2	0	0.935	0	0	0.950^a^
T1b	9	2		5	2	
T2a	28	6		11	6	
T2b	62	15		28	15	
T3	16	5		7	5	
Symptoms	51/117	13/28	0.786	27/24	13/28	0.957^a^
PS	0.179 ± 0.275	0.254 ± 0.087	**<0**.001*	0.247 ± 0.092	0.254 ± 0.087	0.674^a^

Data are expressed as *n* (%) or mean ± standard deviation; BMI, body mass index, TB, total bilirubin; ALT, alanine aminotransferase; AST, aspartate aminotransferase; ASA, American score of anesthesiologists; PS, propensity score. * and bold values indicate statistically significant *P*-value (*P* < 0.05).

^a^ Pearson Chi-square tests.

### Clinical characteristics of 79 matched patients after PSM

The 28 patients who underwent REC were matched with 51 of the 117 patients who underwent OEC. The propensity scores suggested no bias in the matched groups (REC group vs. OEC group: 0.254 vs. 0.251, *P* = 0.647). Figure 4C shows a dot plot of the covariate balance in terms of the standardized mean difference (SMD) for all the individual covariates; the covariate balance improved in the matched data. A line plot of the SMD and the SMD of all confounders is shown in Figure 4B; the standard deviation of the PS decreased after matching. The clinical characteristics of the matched patients were compared, and no significant differences were shown between the groups, considering all 10 variables (Table 1).

### Operative outcomes of 79 matched patients after PSM

Patients who underwent REC had a longer operation time (212.39 ± 73.19 vs. 186.75 ± 66.60 min, *P* = 0.134) and lower amount of bleeding (99.11 ± 115.32 vs. 156.08 ± 242.64 ml, *P* = 0.467) than patients in the OEC group. According to the T stage, lymph node metastasis, and distant metastasis of patients with GBC, as well as re-evaluation of the stage and resectability during the operation, 25 patients underwent wedge resection around the gallbladder fossa (REC group, *n* = 8; OEC group, *n* = 17), 42 underwent bisegmentectomy of segments IVb and V (REC group, *n* = 15; OEC group, *n* = 27), 11 underwent right hemihepatectomy (REC group, *n* = 5; OEC group, *n* = 6), and 1 underwent Right hepatic trisegmentectomy (REC group, *n* = 0; OEC group, *n* = 1) with no significant difference between the two groups (*P* = 0.762). Additionally, the mean number of lymph nodes retrieved was 4.89 ± 2.78 in the REC group and 4.59 ± 2.22 in the OEC group, with no significant difference between the two groups (*P* = 0.828). There were 5 patients with liver cirrhosis diagnosed by pathological examination after operation, and the evaluation of liver function was child A (REC group, *n* = 2; OEC group, *n* = 3, *P* = 0.826). Furthermore, the mean visual analogue scale score after the operation was significantly lower in the REC group immediate postoperatively (3.68 ± 2.09 vs. 4.73 ± 1.85, *P* = 0.008), on postoperative day 1 (2.96 ± 1.75 vs. 3.69 ± 1.41, *P* = 0.023), and on postoperative day 2 (2.36 ± 1.55 vs. 2.92 ± 1.21, *P* = 0.031). However, the time to first ambulation, time to drainage tube removal, and postoperative stay were significantly lower in the REC than OEC group (49.57 ± 16.51 vs. 57.47 ± 16.17 h, *P* = 0.043; 7.86 ± 5.28 vs. 11.08 ± 7.65 days, *P* = 0.038; and 10.11 ± 5.74 vs. 13.65 ± 8.48 days, *P* = 0.037, respectively). But hospital costs were significantly higher in the REC than OEC group (86,174 ± 12,148 vs. 70,400 ± 31,612 yuan, *P* < 0.001). In addition, the incidence of postoperative morbidity and mortality (Clavien–Dindo I–II and III–IV complications) was not statistically significant between the groups (Table 2).

**Table 2:** Operative outcomes according to operation type after propensity scoring match.

Variables	Control (*n* = 51)	Treated (*n* = 28)	*P*-value
Operation time, min	186.75 ± 66.60	212.39 ± 73.19	0.134^b^
Intraoperative blood loss, ml	156.08 ± 242.64	99.11 ± 115.32	0.467^b^
Hepatic resection site			
Wedge resection around the gallbladder fossa	17	8	0.762^a^
Bisegmentectomy of segments IVb and V	27	15	
Right hemihepatectomy	6	5	
Right hepatic trisegmentectomy	1	0	
Number of lymph nodes retrieved	4.59 ± 2.22	4.89 ± 2.78	0.828^b^
VAS score			
Immediate postoperative	4.73 ± 1.85	3.68 ± 2.09	**0.008**^b^*
POD1	3.69 ± 1.41	2.96 ± 1.75	**0.023**^b^*
POD2	2.92 ± 1.21	2.36 ± 1.55	**0.031**^b^*
First ambulation time, h	57.47 ± 16.17	49.57 ± 16.51	**0.043**^b^*
Drainage tube removal time, days	11.08 ± 7.65	7.86 ± 5.28	**0.038**^b^*
Postoperative morbidity (%)	11 (21.6%)	3 (10.7%)	0.227^a^
Clavien–Dindo I–II (%)	6 (11.8%)	2 (7.1%)	0.515^a^
Clavien–Dindo III–IV (%)	5 (9.8%)	1 (3.6%)	0.317^a^
Postoperative mortality (%)	2 (3.9%)	0	0.289^a^
POS, days	13.65 ± 8.48	10.11 ± 5.74	**0.037**^b^*
Hospital cost, yuan	70,400 ± 31,612	86,174 ± 12,148	**<0.001**^b^*

VAS, visual analog scale; POD, postoperative day; POS, postoperative hospital stay; Data are expressed as *n* (%) or mean ± standard deviation; * and bold values indicate statistically significant *P*-value (*P* < 0.05).

^a^ Pearson Chi-square tests.

^b^ Mann–Whitney *U* test (Wilcoxon rank sum W test).

44 patients in OEC group were T1b–T2b, of which 13 patients were diagnosed as GBC by frozen section examination after cholecystectomy, and then underwent wedge resection of liver. The remaining 31 patients underwent extended cholecystectomy directly. 23 patients in the REC group were T1b–T2b, of which 5 were diagnosed as GBC by frozen section examination after cholecystectomy, and then underwent robotic wedge resection of liver. The remaining 18 patients underwent robotic extended cholecystectomy directly.

46 patients in OEC group were T2–T3. Among them, 39 patients were treated with Gimeracil and Oteracil Potassium capsule combined with Gemcitabine chemotherapy after operation, 4 patients refused chemotherapy, and 3 patients could not tolerate chemotherapy. 26 patients in REC group were T2–T3, of which 21 patients were treated with Gimeracil and Oteracil Potassium capsule combined with Gemcitabine chemotherapy after operation, 4 patients refused chemotherapy, and 1 patient could not tolerate chemotherapy.

### Long-term survival outcomes of 79 matched patients after PSM

**Figure 6:**
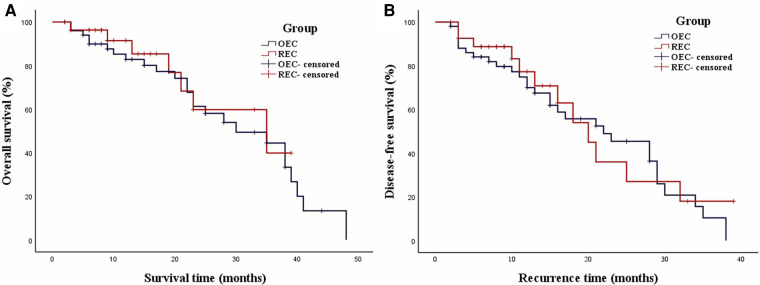
Weighted Kaplan–Meier plot for OS and DFS in the OEC and REC groups. (**A**) The median OS in the OEC and REC groups was 30.0 and 35.0 months, respectively (*P* = 0.619). (**B**) The median DFS in the OEC and REC groups was 22.0 and 20.0 months, respectively (*P* = 0.684).

The mean follow-up duration was 20.1 ± 12.6 months in the OEC group and 16.0 ± 10.7 months in the REC group, with no significant difference between the groups (*P* = 0.228). The median OS in the OEC and REC groups was 30.0 months [95% confidence interval (CI), 16.9–43.1 months] and 35.0 months (95% CI, 13.0–57.0 months), respectively (Figure 6A). The 3-year OS rates were not significantly different between the OEC and REC groups (62.8% vs. 75.0%, respectively; *P* = 0.619). The median DFS in the OEC and REC groups was 22.0 months (95% CI, 12.8–31.2 months) and 20.0 months (95% CI, 14.0–26.0 months), respectively (Figure 6B). The 3-year DFS rates were not significantly different between the OEC and REC groups (43.1% vs. 57.2%, respectively; *P* = 0.684).

## Discussion

Because GBC is accompanied by gallstones or inflammation and lacks specific clinical manifestations, its preoperative and intraoperative diagnosis is difficult. Therefore, the use of adequate and effective imaging methods is very important. Ultrasound is the preferred method for diagnosing gallbladder disease because of its simplicity and high sensitivity (15). The resolution of CT for GBC lesions, surrounding tissue and organ invasion, and distant metastasis is significantly higher than that of ultrasound; in particular, enhanced CT thin-section scanning technology has a higher recognition rate for small lesions of early GBC (16, 17). Compared with CT, magnetic resonance imaging combined with magnetic resonance cholangiopancreatography can more sensitively display GBC and its involvement with adjacent organs, more clearly show signs of biliary obstruction caused by involvement of the intrahepatic and extrahepatic bile ducts, and help to accurately assess the extent of local tumor invasion (18, 19). Intraoperative frozen pathological examination is also an important diagnostic method for GBC (20). However, because of the limited scope of intraoperative frozen pathological examination, the entire gallbladder wall cannot be included, and it is difficult to distinguish mucosal dysplasia from focal GBC. The sensitivity of intraoperative frozen pathological detection of cancer cells ranges from 64.0% to 84.2%, and the sensitivity increases with the depth of tumor invasion (21, 22). In this study, for patients with T1b–T2b gallbladder cancer, intraoperative frozen sections were mainly used for diagnosis to determine whether extended cholecystectomy was required. For patients with T3 gallbladder cancer, intraoperative frozen sections were used to judge the tumor margin to achieve R0 resection.

Extended cholecystectomy broadly includes liver resection, pancreaticoduodenectomy, portal vein resection, extended regional lymph node dissection, hepatopancreatoduodenectomy, and even right upper abdominal resection (23–26). The scope of liver resection is mainly determined according to the location of the tumor and the extent of infiltration, and it may include gallbladder and liver wedge resection, liver segment resection, hemihepatectomy, or liver trilobectomy (27, 28). For T1a GBC, cholecystectomy is usually sufficient; in the present study, however, two patients with T1a GBC in the OEC group had severe gallbladder abscesses and required liver wedge resection. Nevertheless, the optimal surgical method for T1b GBC remains controversial. Lee et al. found no statistically significant difference in the prognosis between extended cholecystectomy and simple cholecystectomy (27). Therefore, they proposed that radical treatment by simple cholecystectomy can meet the needs of patients with stage T1b GBC. However, there are differing opinions on this issue (29, 30). Because of the particularity of the anatomy of the gallbladder area (31), the gallbladder tissue lacks protection, and tumor cells can metastasize through the blood supply and lymphatic system. The range of GBC micrometastasis can even invade the liver tissue 16 mm from the gallbladder bed. Considering the previous literature and actual clinical experience, our institution prefers the use of liver wedge resection to treat T1b GBC because it meets the principle of a tumor-free technique for surgical treatment. In addition, numerous studies have shown that extended cholecystectomy combined with lymph node dissection can achieve R0 resection for patients with T2 and T3 GBC (32, 33).

In recent years, da Vinci robotic surgeries have been widely used in many fields, because these procedures provide magnified three-dimensional high-definition views as well as motion and tremor filtering. Additionally, the enlarged anatomical structures can reduce unnecessary damage during the operation, especially GBC surgery (34). At the same time, the clear visualization of anatomical structures facilitates safe and effective anastomosis of blood vessels and bile ducts and dissection of lymph nodes. As shown in Table 3 (9–11, 35–39), previous studies have revealed that REC has numerous advantages including a lower blood loss volume, lower complication rates, and a shorter postoperative stay. In addition, among patients with GBC, robotic surgery is not inferior to open surgery in terms of the number of lymph nodes resected. In this study, we found no statistically significant difference in the operation time, blood loss, or number of lymph nodes resected between the REC and OEC groups. However, the patients who underwent REC had a shorter postoperative hospital stay (*P* = 0.037) and less pain (*P* < 0.05).

**Table 3:** Previous reports about the robotic extended cholecystectomy in gallbladder cancer.

Author. Year	*N*	Operation time (min)	Blood loss (ml)	Postoperative stay (days)	Morbidity	Mortality	≥T2 stage	Retrieved LNs
Shen et al. 2012	5	200 (120–300)	210 (50–400)	7 (7–8)	0	0	5 (100%)	9 (3–11)
Khan et al. 2018	11	219 (99–790)	50 (10–200)	4 (2–9)	4 (36.4%)	0	11 (100%)	5 (0–9)
Sucandy et al. 2021	15	222 (151–323)	200 (87–357)	3 (1–8)	2 (13.3%)	0	-	-
Ahmad et al. 2020	10	173 (95–240)	88 (30–200)	4 (2–6)	1 (10.0%)	0	10 (100%)	2 (0–5)
Araujo et al. 2020	3	392 (376–408)	186 (60–312)	3 (3)	0	0	0	4 (3–6)
Goel et al. 2019	27	295 (200–710)	200 (20–700)	4 (2–12)	1 (3.7%)	0	22 (81.5%)	10 (2–21)
Zeng et al. 2018	3	243 (165–530)	175 (50–700)	4 (2–8)	0	0	3 (100%)	6 (1–11)
Byun et al. 2020	13	188 (153–223)	271 (0–569)	7 (5–8)	2 (15.4%)	0	13 (100%)	7 (4–10)
Our study	28	212 (139–285)	99 (0–214)	10 (4–16)	2 (7.1%)	0	26 (92.9%)	5 (2–8)

The oncologic outcome after REC in patients with GBC is an important issue. Most previous studies focused on short-term results. For example, Goel et al. (38) reported that the postoperative complication rate was higher after open radical cholecystectomy than after robotic radical cholecystectomy (1 vs. 15 patients, respectively; *P* = 0.035), with only one patient developing major morbidity following robotic radical cholecystectomy. Few studies have compared the long-term outcomes of OEC and REC for patients with GBC, and the present study is the first to compare the OS and DFS of OEC and REC. However, two studies addressed the long-term outcomes after robotic liver resection in patients with hepatocellular carcinoma. Chen et al. (40) reported that robotic liver resection showed a 3-year DFS rate comparable to that of open liver resection in patients with hepatocellular carcinoma (72.2% vs. 58.0%, respectively; *P* = 0.062). Eric et al. (41) compared laparoscopic liver resection and robotic liver resection for hepatocellular carcinoma and found similar 5-year OS (65% vs. 48%, *P* = 0.28) and DFS (42% vs. 38%, *P* = 0.65) between the two groups. In the present study, the median OS in the OEC and REC groups was 30 and 35 months, respectively, and the median DFS was 22 and 20 months, respectively. All patients with GBC in the REC group achieved R0 resection and showed long-term outcomes comparable to those in the OEC group.

In summary, the short- and long-term results in this study indicate that the use of a robotic surgical system for the treatment of GBC is safe, effective, and feasible compared with OEC. There was no difference in the OS or DFS between the two groups. REC was accompanied by less pain, a shorter postoperative hospitalization time, and more rapid postoperative recovery than OEC. Thus, REC is a suitable minimally invasive procedure for the treatment of GBC. Notably, this study had a limited sample size and did not address all types of extended cholecystectomy. This was also a retrospective study with a short follow-up period. The results of this study therefore need to be further confirmed by large-scale multicenter prospective randomized controlled trials and longer-term follow-up.

## Data Availability

The raw data supporting the conclusions of this article will be made available by the authors, without undue reservation.
